# Somatic Mosaicism in the Human Genome

**DOI:** 10.3390/genes5041064

**Published:** 2014-12-11

**Authors:** Donald Freed, Eric L. Stevens, Jonathan Pevsner

**Affiliations:** 1 Program in Biochemistry, Cellular and Molecular Biology, Johns Hopkins School of Medicine, Baltimore, MD 21205, USA; E-Mail: freedd@kennedykrieger.org; 2 Department of Neurology, Kennedy Krieger Institute, 707 N. Broadway, Baltimore, MD 21205, USA; 3 Department of Psychiatry and Behavioral Sciences, Johns Hopkins School of Medicine, Baltimore, MD 21205, USA

**Keywords:** mutation, somatic, germline, mosaicism, complex disease, retrotransposition, neurodegeneration, aging

## Abstract

Somatic mosaicism refers to the occurrence of two genetically distinct populations of cells within an individual, derived from a postzygotic mutation. In contrast to inherited mutations, somatic mosaic mutations may affect only a portion of the body and are not transmitted to progeny. These mutations affect varying genomic sizes ranging from single nucleotides to entire chromosomes and have been implicated in disease, most prominently cancer. The phenotypic consequences of somatic mosaicism are dependent upon many factors including the developmental time at which the mutation occurs, the areas of the body that are affected, and the pathophysiological effect(s) of the mutation. The advent of second-generation sequencing technologies has augmented existing array-based and cytogenetic approaches for the identification of somatic mutations. We outline the strengths and weaknesses of these techniques and highlight recent insights into the role of somatic mosaicism in causing cancer, neurodegenerative, monogenic, and complex disease.

## 1. Introduction to Somatic Mosaicism

### 1.1. Early Studies of Mosaicism

**Figure 1:**
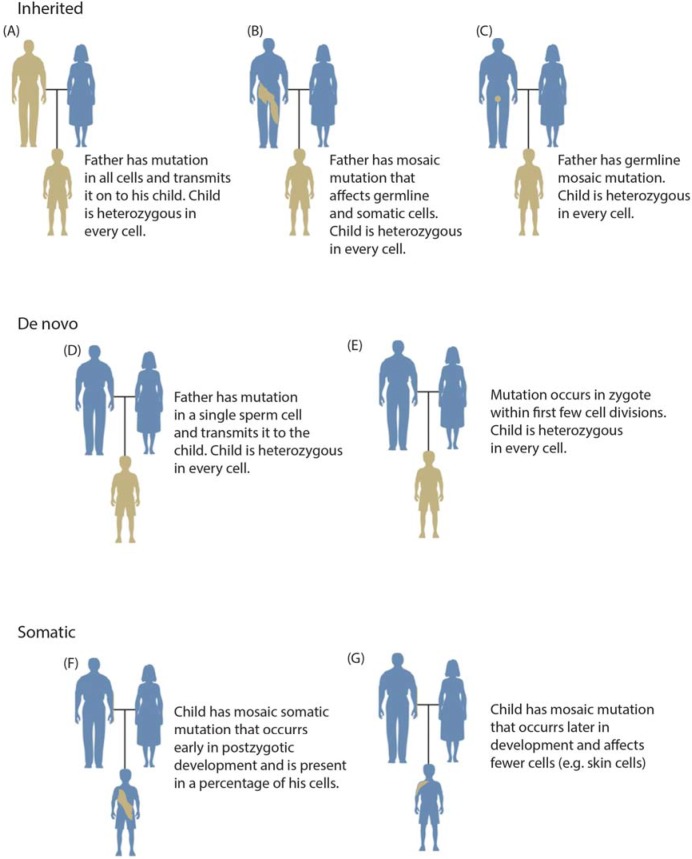
Overview of categories of variation including inherited (panels A–C), *de novo* (panels D,E), and somatic variation (panels F,G). Inherited mutations are always transmitted through the germline (**A**); although a parent may also have a mosaic mutation (this combination of somatic and germline mosaicism is occasionally termed gonadal mosaicism) (**B**); In such cases, a child may inherit the variant as a heterozygous mutation with a more severe clinical phenotype. A parent may also have germline mosaicism that may be inherited by progeny (**C**); *De novo* mutations are operationally defined as genotypes observed in a child but not in either parent. They may originate in a parental germ cell (as may be inferred in a pedigree having multiple affected offspring) (**D**) or postzygotically (**E**); Somatic mutation may occur relatively early in development (**F**) or at any later time throughout the lifespan (**G**), generally affecting fewer cells.

Somatic mosaic mutations are defined as mutations that occur in some cells of the soma of a single individual (Figure 1) [1,2]. The mixture of mutation-positive cells with non-mutated cells results in an individual who is a mosaic, or contains different DNA within different cells of his or her body. Mosaic mutations may be present in the germline or soma; however, typically only mutations in the soma have phenotypic consequences or are detectable by current genotyping methods. Mosaic mutations in germ cells are usually only discovered when they lead to inherited conditions in multiple progeny. *De novo* mutations are operationally defined as mutations found in all cells of an individual but not detected in that individual’s parents (Figure 1d,e) [3]. *De novo* mutations only present in the offspring may occur very early in development; however, this is rare and increasingly sensitive genetic assays are discovering low‑level parental mosaicism in supposedly *de novo* cases (Figure 1b) [4,5].

The role of somatic genetic changes in human health has been considered at least since 1914 when Theodor Boveri recognized that cancers frequently have abnormal karyotypes [6]. Alfred Knudson built upon the work of Boveri and others and in 1971 described a two-hit model of cancer resulting from both an inherited germline mutation and a later somatic mutation [7]. The model of metastatic cancer occurring as a result of multiple mutations in a single cell lineage has remained largely unchanged for over 40 years [8,9].

The scientific community was slower to realize the importance of postzygotic mutational events outside of cancer. In the early 1950s, Barbara McClintock demonstrated the phenotypic importance of somatic transposition in *Zea mays*, and in 1959 Sir Macfarlane Burnet proposed a role for somatic mutation in disease [10,11]. Nonetheless, few studies indicated a role for somatic mosaicism in human health. This changed in the 1970s with the discovery that somatic gene rearrangement creates functional diversity of immunoglobulin and T-cell receptor genes [12,13,14]. Today, it is known that somatic mutations are ubiquitous [15] and have important roles in cancer [9], aging [16,17], neurodegeneration [18], monogenic disease [19,20,21], reversion of inherited disease [22,23,24,25], and numerous neurocutaneous disorders [26].

### 1.2. Categories of Somatic Variation

Somatic variation has been observed at all genomic scales from point mutations to aneuploidies. At the level of whole chromosomes and large chromosomal segments, complex genomic rearrangements occur somatically (as well as in the germline). The loss or gain of entire chromosomes is thought to be caused by errors in chromosomal segregation during anaphase, while non-allelic homologous recombination may cause the loss, gain, or rearrangement of large genomic regions [27,28]. The phenotypic consequences of these events vary considerably based on the size of the event and the genomic region involved.

In many instances, both copies of a chromosome pair (or of a chromosomal segment) are inherited from one parent, a phenomenon termed uniparental disomy (UPD) [29,30]. UPD may involve two copies from a parent that are identical (uniparental isodisomy) or different (uniparental heterodisomy). Either form may disrupt epigenetically imprinted regions (defined as undergoing differential expression depending on the parent of origin), while uniparental isodisomy may also expose two copies of a recessive mutation. One mechanism for the occurrence of UPD involves trisomic rescue in which an extra (third) copy of a chromosome is rejected, producing a diploid cell line in which one parent’s monoploid copy is lost [31]. Frequently, the trisomic rescue is restricted to a fraction of cells in an individual resulting in mosaic trisomy/UPD [32]. UPD may also result from somatic recombination occurring from a reciprocal exchange during mitosis, leading to loss of heterozygosity.

RNA-templated DNA polymerases are another cause of genomic instability. While numerous types of repetitive elements are present in human genomes, only non-long terminal repeat retrotransposons are currently competent for retrotransposition [33]. Successful retrotransposition of these elements is dependent upon functional protein products from long interspersed elements (LINEs). In most somatic tissues, LINEs are epigenetically suppressed; however, these elements escape epigenetic repression during early embryonic development, and their integration into other functional genomic elements occasionally results in disease such as choroideremia (Online Mendelian Inheritance in Man [OMIM] #303100) [34]. Retrotransposition may also occur in somatic tissues with unusual epigenetic states [35].

Low complexity regions, including trinucleotide repeats, are scattered throughout the mammalian genome. Trinucleotide repeats can be hypervariable and expansions of some trinucleotide repeats are the causes of nearly 30 disorders [36,37]. The molecular mechanisms underlying expansion or contraction of these regions are complex and cause these regions to have variable length throughout the body of those afflicted with disease [38,39,40,41,42,43,44].

Small genetic aberrations may be caused by a number of mechanisms. Polymerase errors may result in nucleotide misincorporation or small insertions or deletions in the germline or soma. Over time, DNA will accumulate numerous lesions and DNA polymerization across these lesions is especially error-prone. DNA lesions may be detected and repaired prior to DNA polymerization, but lesion repair may also create single nucleotide variants, or small insertions or deletions [45,46].

In linear mammalian genomes, DNA replication starts at multiple origins with DNA polymerases ε and δ [47,48]. Polymerase ε moves with high processivity 5'–3' along the genome on the leading strand, moving in the same direction as the replication fork. On the lagging strand replication by polymerase δ also proceeds 5'–3' but in the opposite direction as the replication fork, causing replication of that strand to be iterative. This process works well for the majority of the genome, but replication of the lagging strand leads to loss of genetic information at the ends of the chromosome during every replication [49]. This end replication problem is solved in the germline because the ends of chromosomes, telomeres, are protected by repetitive DNA which is synthesized by a dedicated RNA-templated DNA polymerase called telomerase [49]. However, telomerase is not usually expressed in somatic tissues, likely as a method of protection against malignant transformation, and decreased telomere length is a form of somatic variation.

### 1.3. Mosaicism during Development

A defining characteristic of mosaic mutations is that they occur postzygotically and are inherited by all subsequent cells in their lineage (Figure 1). Somatic errors in chromosomal segregation in early development induce an extraordinarily high rate of aneuploidy. Fifteen to 20% of clinically recognized pregnancies result in spontaneous abortion, and half of these are attributed to aneuploidy [29]. A review of 36 published studies showed that of 815 human preimplantation embryos, only 177 (22%) were diploid while 73% were mosaic [50]. In most cases, these were diploid-aneuploid mosaic embryos, having one or more diploid cells as well as other cells that were haploid or polyploid for a particular chromosome. Mitotic errors could account for the high rate of chromosomal mosaicism.

Due to the exponential rate of growth during development, somatic mutations must occur early in development to have phenotypic effects over large portions of the body. Severe somatic mutations, which would be embryonic lethal if inherited, have a short window during development in which they must occur to be observed in adults [19]. If these severe mutations occur early in development, they will be embryonically or prenatally lethal; occurring later in development they may have little or no obvious phenotypic effect.

Mutations that alter cellular growth do not necessarily have to occur within such a short developmental window. Inactivating mutations in genes encoding tumor suppressors or activating mutations in oncogenes may have functional consequences regardless of when they occur, as evident from their known roles in cancer. On the other hand, somatic growth-retarding mutations, such as inactivating mutations in oncogenes or certain cyclins, are unlikely to have phenotypic effect in adults regardless of when they occur in development as the total number of cells containing the mutation is likely to be small.

Somatic mutations are thought to occur in all cells during replication. On average, 50 mutations occur in microsatellite regions during every mitotic division of a given cell [15]. Mutations in microsatellites and other regions of the genome, assessed by either single-cell or deep sequencing, can then be used to infer cell lineage trees [51]. To date, the most successful lineage tracing experiments have made use of increasingly sophisticated microscopy techniques [52]. However, microscopy-based approaches have practical and technological barriers such as the requirement that non-transgenic cells must be monitored over time. Recent advances in whole genome amplification (WGA) and second-generation sequencing offer genetic‑based approaches that do not have the same limitations. Already, these techniques have been used to provide a detailed view of the genetics of cancer metastasis [53,54].

### 1.4. Mosaicism across the Body

By definition, somatic mosaic mutations affect only a subset of cells within an individual (Figure 1). This is most easily visible in monogenic mutations affecting pigmentation patterns. While such patterns may be mistaken for stochastic X chromosome inactivation or autoimmune response, somatic mutation is generally localized over a small portion of the body and in many cases occurs along lines of Blaschko [55]. To date, almost all non-cancerous somatic mutations characterized at the molecular level result in visible abnormalities, usually involving hypertrophy (cellular overgrowth) or abnormal pigmentation [26,55]. Some of our inability to identify mutations that do not result in visible phenotypes is practical; during dissection it is difficult to distinguish affected from unaffected tissue. However, due to the current emphasis on visible phenotypes, few data are available on the extent to which non-visible somatic mutations influence important biological processes.

An important consideration is that somatic mutations occur in varying cell types and tissues as well as different developmental stages. This raises the possibility that a specific mutation may vary in its clinical importance depending on where the mutation occurs across the body. Mutations in *GNAQ* provide an example. We identified p.Arg183Gln mutations in *GNAQ*, encoding the G protein alpha subunit Gαq, as the cause of both Sturge-Weber syndrome (OMIM #185300) and port-wine stain birthmarks (OMIM #163000) [56]. Port-wine stains are non-syndromic vascular abnormalities, while the Sturge-Weber syndrome is a severe neurocutaneous disorder, although both conditions likely affect some of the same cell types (e.g., endothelial cells). The milder phenotype of the birthmarks could result from a later developmental origin of the mutation during fetal development. The identical p.Arg183Gln mutation in *GNAQ*, when occurring in melanocytes later in life, is a frequent driver mutation in uveal melanoma (OMIM #155720), highlighting the importance of both the location and timing of the mutation. p.Arg183Gln mutations in different cell types and developmental stages could have different phenotypic consequences, if any [57].

Other mosaic mutations also differ in their clinical importance based on cell or tissue-specific involvement. McCune-Albright syndrome (OMIM #174800) is characterized by increased function of endocrine glands, sexual precocity, café-au-lait macules, and fibrous dysplasia. These symptoms can vary considerably based, in part, on the bodily extent of the mutation [58]. Like Sturge-Weber syndrome, this disorder is caused by somatic activating mutations in a gene encoding a G protein alpha subunit (*GNAS* encoding Gαs). Expression of this gene highlights another dimension of mosaicism. *GNAS* is expressed biallelically through most of the body, but the maternal allele is imprinted in particular tissues such as the pituitary. The disorders progressive osseous heteroplasia (OMIM #166350) and pseudopseudohypoparathyroidism (OMIM #612463) result from loss of function mutations in the paternal allele of *GNAS* [59].

**Figure 2:**
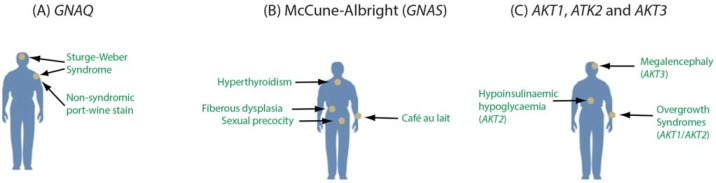
Tissue-specific effects of mutations in *GNAQ* (**A**); *GNAS* (**B**); and *AKT1*, *AKT2*, and *AKT3* (**C**). Constitutively activating mutations in *GNAQ* may lead to either Sturge-Weber syndrome, nonsyndromic port-wine stains, or uveal melanoma (**A**). Somatic activating mutations in *GNAS* lead to McCune-Albright syndrome, which may involve variable hyperthyroidism, *café au lait* macules and sexual precocity (**B**). Activating mutations in all three of the *AKT* genes cause cellular overgrowth phenotypes with mutations in* AKT2* also implicated in abnormal insulin signaling (**C**).

Somatic mutations in three *AKT* genes also have cell-specific effects [60,61,62]. Somatic *AKT1* mutations are associated with somatic breast cancer, colorectal cancer, and ovarian cancer as well as the Proteus syndrome. The *AKT2* gene is expressed selectively in insulin-responsive tissues and mutations are associated with diabetes. Somatic mutations in *AKT3* cause Megalencephaly-polymicrogyria-polydactyly-hydrocephalus syndrome 2 (OMIM *615937). Given the localized nature of somatic mutations in *AKT* discovered to date, it is likely that mutations in these genes occurring outside of vulnerable cell types have few effects. These examples highlight the complex interaction of localized somatic mutation with tissue or cell-specific gene expression and signaling pathways (Figure 2).

Numerous studies have aimed to assess the prevalence of mosaic alterations in tissues of apparently normal individuals. Reanalysis of data from multiple large genome-wide association studies have determined that the number of detectable mosaic events rises sharply after age 50. Furthermore, individuals with increased numbers of mosaic events have higher risk for developing cancer [63,64]. While this measured increase of mosaicism may be due to increased rates mutation rates in elderly individuals, it is much more likely that these events are the result of clonal expansion and positive selection within the stem cell niche or decline in the total number of hematopoietic stem cell progenitors later in life. Notably, increased rates of mosaicism in apparently normal tissues have been linked to poorer prognosis in individuals with ovarian cancer [65].

Studies of twins have demonstrated that post-zygotic mutations may be phenotypically important. Notable examples are monozygotic twins who are discordant for phenotypic sex due to mosaic loss of chromosome Y [66,67]. Numerous examples of monozygotic twins exist where either the presence [68,69] or severity [70] of disease is discordant between twin pairs due to variable proportions of mosaic cells.

Studies of multiple tissues of apparently normal individuals have also found evidence for mosaic events. Analysis of CNVs using hybridization of DNA from multiple tissues of three apparently normal individuals to bacterial artifical chromosome arrays found evidence for six somatic CNVs [71]. Higher resolution examination of a total 33 tissues from six individuals using array comparative genomic hybridization found evidence for 73 high-confidence mosaic CNVs, although a majority of high-confidence events (54/73) were found in one of two particular tissues [72]. It has been noted that induced pluripotent stem cells (iPSC) frequently contain CNVs which may cause genomic instability inheritent to the process of immortalization. Abyzov* et al.* performed a detailed study of this phenomenon and concluded that almost half of CNVs present in iPSC lines can be found in the parental fibroblasts. Furthermore, they conclude that approximately 30% of all fibroblasts in their sample contain some mosaic CNVs [73].

While experimentation with bulk tissues has shown that somatic mosaicism occurs frequently in normal populations, the combination of DNA from many cells limits the ability of an assay to detect mosaic events unique to single or few cells. As a result, sequencing of single-cells has been recently used to assay mosaicism in normal tissues. These methods have been used to sensitively reexamine conclusions regarding the extent of mosaicism in the brain. Previous reports had indicated that up to 33% of neuroblasts were aneuploid while up 80 retrotransposon insertions occur per neuron [74,75,76,77]. Single-cell experiments of the same phenomena have shown that large copy-number variants occur in over 14% of neurons but whole chromosome aneuplodies and retrotransposition events are relatively rare [78,79,80].

Single-cell studies have also been used to investigate the extent to which mosaicism occurs in early development. It has been known since 1983 that chorionic villus sampling may indicate the presence of a trisomy, while the fetus is diploid without the presence of mosaicism, a condition termed confined placental mosaicism [81,82,83]. Single-cell studies of young embryos cultured* in vitro* also demonstrate that chromosomal aneuploidies are common and were found in 83% of tested embryos [84]. While it is likely that many aneuploid embryos are unlikley to result in viable pregnancies, recent advances in prenatal testing allow for the sensitive and specific detection of numerous trisomies by sequencing of circulating fetal DNA from maternal plasma [85].

## 2. Detection of Somatic Mosaicism

### 2.1. Technical Considerations

Almost every type of genetic variation has been implicated as a source of somatic variation including expansion of trinucleotide repeats, point mutation, copy-number loss/gain, uniparental disomy, mitotic recombination, aneuploidy, translocation, and retrotransposition [39,40,41,43,44,69,77,79,86,87,88,89,90,91,92,93,94]. The techniques summarized below vary widely in their ability to detect specific types of somatic variation and more specialized techniques exist for the sensitive detection of some types of variation.

A primary consideration during the analysis of mosaic samples is the purity of the dissection from tissue samples. The presence of normal cells in affected tissue significantly decreases the ability of downstream analyses to detect mosaic alterations. This problem can be compounded by the prevalence of cellular migration during development in some tissues. Thus, in a tissue affected by a somatic mutation, two neighboring cells may both be affected if they share a common lineage from the mutated cell. Alternatively, cellular migration could cause neighboring cells to originate from distinct precursors with only one cell affected. Cellular migration can place an important biological constraint on the visible frequency of driver somatic mutations in affected tissues (e.g., in the brain) [3,95,96].

**Figure 3:**
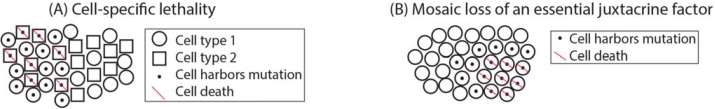
Cell death may reduce the total number of cells harboring somatic mutation. Mosaic mutations may cause cell-type-specific lethality (**A**); Mosaic loss of an essential juxtacrine signaling factor may cause localized death of cells that are not adjacent to unaffected tissues (**B**).

While contamination of normal cells is known to decrease the observed frequency of mosaic mutations, other mechanisms may decrease the detectable fraction of mosaic cells within a sample. Two possibilities are cell-type specific lethality and mosaic absence of essential juxtacrine or paracrine signaling factors. Cellular signaling pathways are known to have cell-type specific effects raising the possibility that a mosaic mutation may be lethal in only one type of cell within a tissue (Figure 3a). Furthermore, some paracrine or juxtacrine signaling factors are essential for cell viability [97,98,99]. Mosaic loss of these factors could result in affected tissue that is dependent upon surrounding normal tissue for survival, reducing the total number of mutant cells (Figure 3b).

In Sturge-Weber affected tissues, we detected *GNAQ* mutant allele frequencies between 1% and 18% [56]. Other studies using similar techniques have detected mutant allele frequencies of 1%–47% [61], 3%–30% [100], and 3%–35% [60] for causative mutations in individuals with Proteus syndrome (OMIM #176920), CLOVE (Congenital Lipomatous Overgrowth, Vascular anomalies, and Epidermal nevi) syndrome (OMIM #612918), and hemimegalencephaly (e.g., OMIM #615937), respectively. Such relatively low allele frequencies are likely explained by the presence of low proportions of affected cells in a given tissue due to cellular migration or impure dissection. However, the occurrence of mosaic cell death due to either cell-specific lethality or loss of essential signaling factors should be considered.

In second-generation sequencing experiments, sequencing and mapping errors are a major concern, as some portions of the genome are known to be prone to false-positive variant calls [101]. Recent improvements in sequencing chemistry have lowered the frequency of sequencing errors. However, biased errors in sequencing are still problematic for the detection of somatic variation, especially when the mutant allele frequency may be close to the technology’s inherent error rate. Generally, ultra-high depth sequencing (>500 reads) of normal and affected tissues will permit detection of these errors. However, exploratory studies generally do not reach this level of depth. It is likely that without validation, these errors are a source of false positives in somatic variation databases. Comparing suspected somatic mutations across multiple tissue types from multiple individuals to estimate local error profiles may be a possible solution to this problem [102].

### 2.2. Cytogenetics

Microscopy-based methods allow for the detection of large mosaic events in single cells. Early cytogenetic methods for identifying extra or fewer chromosomes involved counting condensed metaphase chromosomes under a microscope [103]. Later methods using Giemsa staining and other dyes produced unique chromosomal bands allowing for the identification of intra- and interchromosomal translocations, duplications, deletions, and large structural rearrangements. However, banding techniques can only resolve aberrations larger than 3–10 Mb [104]. Other methods, such as fluorescent* in situ* hybridization (FISH), label a specific region of the genome by hybridization of a fluorescent probe allowing for the detection of deletions and some duplications [105]. Variations in this methodology exist using multiple probes of different color to detect several unique fragments at a time (e.g., multicolor FISH). These methods are able to achieve resolutions below 100 kilobases or, in some cases, as few as several kilobases [106]. Potential probe binding to off-target regions is a major consideration in most FISH experiments and adequate controls are required to confirm locus specificity [106]. Variants on classical FISH methods continue to be developed which promise to increase the ability of fluorescent probes to detect small chromosomal abnormalities across increasingly large portions of the genome [106,107]. In combination with high-throughput techniques, these approaches may be used to screen large numbers of cells from a single individual allowing for the detection of low levels of mosaicism.

### 2.3. Genome-Wide Arrays

Comparative genomic hybridization (CGH) is a technique in which fluorophore-labeled DNA from a control and test individual are hybridized to a metaphase reference chromosome [108]. The ratio of fluorescence emission is then measured to allow for the detection of duplication or deletions. A ratio of 1:1 indicates that both samples of DNA carry the same copy number while deviations from this ratio indicate a copy number variant [109].

Two principal array-based techniques that have emerged as alternatives to CGH are array CGH (aCGH) and single nucleotide polymorphism microarrays (SNP microarrays) [110,111,112]. Similar to CGH, both aCGH and SNP microarrays have the ability to detect changes in copy number over large regions of the genome. SNP microarrays further have the ability to genotype individuals at the probed sites, which may be useful in the detection of low-level somatic events [113]. Array-based approaches offer increased sensitivity over the entire genome for small CNVs relative to genome-wide microscopy-based approaches. aCGH and SNP microarray analysis can resolve regions less than 100 kb in size. However, the sensitivity of array-based approaches for somatic CNVs is dependent on having at least 5%–10% of the cells assayed containing the genetic variant. For larger CNVs affecting a smaller fraction of cells, microscopy-based approaches are more sensitive.

In both aCGH and SNP microarrays, deviations in relative probe intensities indicate deletion or insertion events. Normalized probe intensities are commonly reported as log-R ratios, with higher intensities indicating insertions while lower intensities indicate deletions. For SNP microarrays, the relative intensities of the two probes (one specific to each allele) at a locus is informative, and normalization of these intensities is measured as a B-allele frequency. For normal diploid tissues, B-allele frequencies approximate 0.0, 0.5, and 1.0 for AA, AB, and BB genotypes, respectively, while log-R ratios approximate 0 indicating no copy number change.

The hybridization of genomic DNA to microarrays is inherently noisy and can be subject to large batch effects [114]. Furthermore, individual probes or even whole arrays may have errors caused by faulty manufacture. Together these artifacts make the detection of statistically significant mosaic CNVs difficult, but many software packages detect these events. Numerous tools use hidden Markov Models (HMMs) to integrate B-allele frequency and log-R ratio information for the detection of mosaic events, including PennCNV-2, GPHMM and MixHMM [115,116,117]. gBPCR uses an approach similar to the Bayesian Piecewise Constant Regression for the detection of mosaic abnormalities but has a long runtime per sample [118]. We developed triPOD which uses multiple algorithms for the detection of mosaic events and is unique in that it utilizes parental genotypes allowing for more sensitive detection of haplotype-specific mosaic abnormalities [113].

### 2.4. Second-Generation Sequencing

Second-generation sequencing techniques have revolutionized human genetics in the last decade. Sequencing is performed either on single cells, a discrete number of cells, or bulk tissue. In the typical sequencing experiment, DNA is extracted from the input material and is fragmented, size-selected, and sequenced to produce strings of inferred nucleotides and their respective quality scores [119]. This information is used to align the sequencing reads to a reference genome. Differences between the aligned reads and the reference can be used to infer genetic variants including single-nucleotide variants or polymorphisms (SNVs or SNPs), insertions, deletions, translocations, and retrotransposition events. Furthermore, the total number of reads aligned to certain regions of the genome can be used to infer copy-number changes [120,121]. Numerous variations on this basic approach exist and here we will discuss the methods most applicable for the detection of mosaic events.

Somatic genetic variants have been discovered via whole-exome or whole-genome sequencing of bulk tissue from paired affected and unaffected portions of the body [56,60,61,100]. Whole-exome sequencing relies upon an oligonucleotide bead or array-based enrichment of DNA fragments corresponding to exonic regions to reduce the representation of sequence from noncoding regions of the genome [122,123,124]. At similar depth, exome and whole-genome sequencing are considered to have similar sensitivity for most pathogenic SNVs and small insertions or deletions. Whole-exome sequencing is considered less sensitive for the identification of medium to large insertions or deletions or the detection of copy-number changes by analysis of read depth due to introduced biases. However, exome sequencing experiments are typically performed at higher depth due to the lower cost of the method.

Numerous software packages allow the identification of somatic variants from these data. Somatic variant callers typically evaluate second-generation sequence data from paired tumor/normal (or other affected/unaffected) samples. Examples include VarScan2 [121], SomaticSniper [125], JointSNVMix [126], Strelka [127], and MuTect [128]. After removal of low-quality reads, sequences are aligned to a reference genome to generate aligned binary sequence alignment/map (BAM) files [129]. At least three approaches have been employed for the detection of somatic SNVs and small insertions or deletions. (1) Allele frequencies can be compared. For example, VarScan2 performs pairwise comparisons of base calls and normalized sequence depth at each position, accounting for factors such base quality scores, coverage and variant allele frequencies; (2) Bayesian comparison of joint diploid genotype likelihood can be estimated for both samples. The SomaticSniper algorithm calculates the statistical significance of all somatic variants at positions above a minimum threshold of coverage using this method; (3) Other Bayesian approaches have been applied. For example, Strelka models the normal sample as germline variation plus noise, while the affected sample includes noise along with germline and somatic variation. Other types of somatic variation may be detected from bulk sequencing. Tools such as VarScan2, ADTeX, Control-FREEC, SomatiCA, and LUMPY may be used for the detection of somatic CNVs or structural variants [121,130,131,132,133].

Besides variant identification, quantification of the fraction of cells affected by particular somatic changes provides a better understanding of the extent of the mosaic mutation and the period during development at which it occurred. Several tools have been developed to deconvolute somatic mutations into distinct populations as reviewed by Yadav and De and Ding *et al.* [134,135]*.*

An alternative approach to sequencing bulk tissue is sequencing single cells or small numbers of cells. As single or hundreds of cells contain very little DNA, most experiments utilize multiple displacement amplification (MDA) or PCR based methods to amplify genomic DNA. Amplification can greatly increase the total amount of available DNA for sequencing at the expense of introduced biases such as allele dropout and chimeric amplification of genomic fragments [79,136,137,138]. Despite these introduced biases, amplification and subsequent second-generation sequencing or array-based analysis of single cells has been used to reliably find somatic copy number variation and retrotransposition events within the human brain as well as to map cell lineage within a bulk tumor dissection [53,54,79,139]. Numerous groups have also used single-cell techniques to discover SNVs or indels in single cells, however, allelic dropout and chimeric amplification are more problematic for these analyses as biases can be reduced for analysis of CNVs by increasing bin sizes but are more difficult to account for in analysis of SNVs [140,141,142].

## 3. Somatic Mosaicism in Disease

### 3.1. Cancer and Aging

**Figure 4:**
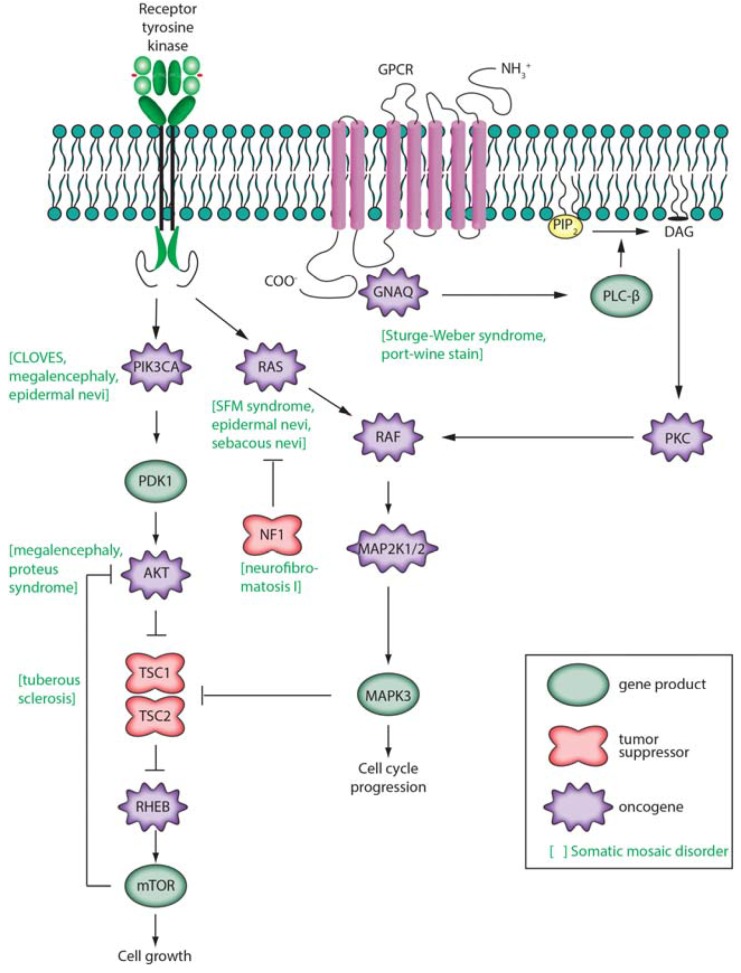
Three intracellular signaling pathways are shown schematically. (**At left**), receptor tyrosine kinase activity leads to activation of PIK3CA, AKT, and mechanistic target of rapamycin (mTOR) [187,188]. mTOR participates in complexes (TORC1, activated by RHEB; TORC2, inhibited by RHEB) that regulate cell growth, proliferation, survival, and cell cycle progression. This pathway includes genes that are frequently mutated in tumors such as *PIK3CA* and *PTEN* (not shown); (**At center**), secreted growth factors bind to receptor tyrosine kinase receptors on the cell surface leading to activation of the low molecular weight G protein Ras and subsequent activation of Raf, MEK 1/2, and ERK 1/2 (official gene symbols *MAPK3*, *MAPK1*); (**At right**), a G-protein coupled receptor (GPCR) pathway is shown [189,190]. Ligands such as vasopressin, endothelin, glutamate, or norepinephrine bind to a GPCR. When bound by ligand, the receptor activates a G protein alpha subunit such as Gαq that binds and hydrolyzes GTP. This leads to activation of phospholipase Cβ producing inositol 1,4,5-triphosphate (IP_3_) and membrane-associated diacylglycerol (DAG). DAG, through activation of protein kinase C, may activate the Raf/MEK/ERK pathway. IP_3_ may bind to an IP_3_ receptor activating calcium signaling pathways (not shown). Other G protein α subunits (such as Gαs encoded by *GNAS*) activate membrane-bound adenylate cyclase, producing cyclic AMP (cAMP) that activates protein kinase A (not shown).

The relationship between somatic mutation and cancer has been extensively reviewed elsewhere [9,17,87,143,144,145] and comprehensive lists of known oncogenes or tumor suppressors or genes significantly and recurrently mutated in cancer have been previously described [9,87]. Cancer has been described as having six hallmarks: proliferative signaling, evading growth suppressors, resisting cell death, enabling replicative immortality, induction of angiogenesis, and inactivating invasion and metastasis [146]. Driver gene mutations are defined as conferring a selective growth advantage in tumor cells [9]. This may be achieved by elevating the activity of growth factors and/or their receptors, but more commonly driver mutations constitutively activate intracellular signal transduction cascades. Three of these are depicted in Figure 4 (in simplified form): Ras/Raf/MEK/ERK, Ras/PI3K/PTEN/Akt/mTOR [147], and *GNAQ*. These pathways contain both oncogenes (*RAS*, *RAF*, *MEK*, *PIK3CA*, *AKT*, *GNAQ*) and tumor suppressor genes (*NF1*, *PTEN*, *TSC1*, *TSC2*). For example the *RAS* family of oncogenes were the first oncogenes to be identified in cancer. Comprised of *HRAS*,* KRAS* and *NRAS*, activating mutations in these genes occur in approximately 20% of all cancers [148]. Germline variants are also well known to contribute to cancer morbidity [149,150,151]. Frequently, these variants affect proteins involved in DNA repair, highlighting the role of somatic mutations in tumorigenesis [152,153,154,155].

In common solid tumors, ~95% of protein altering mutations consist of single base substitutions, >90% of which are missense mutations, <8% are nonsense mutations, and <2% affect splice sites or untranslated regions [9]. Relatively large numbers of somatic mutations occur in tumors that are associated with mutagens such as ultraviolet light and cigarette smoke. For example, in non-small cell lung carcinomas the average mutation frequency is greater than ten-fold higher in smokers compared to those who never smoke [156].

Large-scale projects and databases have been developed to provide comprehensive catalogues of somatic mutations found in cancer [157,158]. COSMIC (Catalogue Of Somatic Mutations In Cancer) includes information on more than 1.6 million mutations from nearly 1 million cancer samples and includes various types of mutations (fusions, genomic rearrangements, whole genomes, and copy number variants) [157].

The combination of well-characterized somatic mutation databases and low-cost sequencing technologies may lead to improved patient outcomes in the near future. Biopsied tumors may be screened rapidly for putative driver mutations based on cancer type, informing treatment. Furthermore, once a cancer is in remission, tumor-specific DNA may be assayed at low cost with ultra-sensitive second-generation sequencing-based techniques [159]. These advances will likely improve prognosis for millions of cancer patients within the next decade.

The primary risk factor for cancer is age, and cancers offer insight into age or mutagen-associated mutational processes [160]. Somatic mutations have long been suspected to be an important part of the molecular mechanism of aging, and accumulation of DNA lesions and mutations occurs in both the germline and soma over time [63,64,161,162]. By chance, these mutations may result in malignant transformation, apoptosis, or otherwise hampered cellular function. As visible in cancers, the characteristics of acquired mutations differ by tissue type and are dependent upon environmental exposure [9]. Furthermore, frequently dividing stem cells and frequently transcribed genomic regions have different patterns of mutation that are cell-type specific.

In both mouse and human, increased rates of somatic mutation and numbers of DNA lesions due to either error-prone DNA polymerases or faulty DNA repair mechanisms cause cancer predisposition, early aging, and neurodegenerative phenotypes [17]. Increased rates of somatic mutation in the nuclear genome cause cancer predisposition, likely due to increased rates of mutation in somatic stem cell populations. This has been demonstrated in transgenic mice whose processive DNA polymerases lack proofreading. Notably, mice with mutated polymerases δ and ε develop distinct cancers but do not demonstrate premature aging phenotypes [163,164,165]. While these mice may not live long enough to demonstrate early aging phenotypes, their predisposition towards the development of cancer demonstrates a strong link between cancer and somatic mutation.

Mutations in genes affecting other pathways demonstrate a strong relationship between somatic mutations and aging. Mice with error-prone mitochondrial polymerases demonstrate a premature aging phenotype without cancer predisposition, although subsequent data by some of the same authors demonstrate that mitochondrial point mutations are unlikely the primary cause of aging in normal mice [166,167]. Individuals with defects in DNA repair also demonstrate symptoms of progeria. Cockayne syndrome (OMIM #216400) is caused by defects in transcription-coupled exonucleotide repair leading to an early aging phenotype combined with intellectual disability and neurodegeneration without noted predisposition to development of cancer [168]. Mutations in the genes encoding RecQ helicases cause Werner syndrome (OMIM #277700) and Rothmund-Thomson syndrome (OMIM #268400) [169]. The most prominent phenotype of individuals affected by these diseases is premature aging, although these individuals are also predisposed to developing cancer [169]. Bloom Syndrome (OMIM #210900) is notable in that it is also caused by mutations in a RecQ helicase-like protein and also increases cancer incidence, but does not appear to result in progeria. Mutations in numerous other genes are known to cause cancer predisposition. One such example is *BUB1B*. Loss of BUB1B protein function leads to premature chromatid separation and mosaic variegated aneuploidy syndrome 1 (OMIM #257300) typically resulting in cancer predisposition and intellectual disability [170].

Cancer is associated with many genomic changes. Large chromosomal changes occur in a variety of noncancerous conditions. An example is Pallister-Killian syndrome (OMIM #601803) is a dysmorphic condition caused by mosaicism for tetrasomy 12p. Affected individuals display tissue mosaicism, typically with apparently normal karyotypes from lymphocytes but 47 chromosomes in skin fibroblasts and chorionic villus and amniotic fluid cells. The extra chromosome is an isochromosome for a portion of chromosome 12p. In several cases hexasomy of chromosome 12p has been observed.

### 3.2. Neurodegenerative Disease

Somatic mutation is suspected to have a role in neurodegenerative disease [17,18]. As in cancer, mutations in genes directly involved in DNA repair are implicated in neurodegenerative diseases such as ataxia-telangiectasia (OMIM #208900) and ataxia-ocular apraxia 1 (OMIM #208920) [16,169,171,172,173,174]. These neurodegenerative phenotypes are likely caused by an increase of somatic mutation in the nervous system leading to cellular dysfunction, indicating a possible role for somatic changes and DNA lesions in age-related related neurodegenerative disorders.

There is evidence that mosaic mutations or accumulated damage to other macromolecules play a role in Alzheimer’s disease (OMIM #104300) and Creutzfeldt-Jakob disease (CJD) (OMIM #123400). Alzheimer’s disease is characterized by the accumulation of β-amyloid (Aβ) plaques while CJD is caused by misfolded protein PRNP [175,176]. Significant incidence of both diseases is attributed to familial risk and causal mosaic mutations have been found in sporadic cases [177,178]. Aβ plaques have long been implicated in the formation of prions and introduction of Aβ plaques into the brains of mice overexpressing Aβ leads to disease progression [179,180,181]. Consistent with the link to prions, the pathology of inoculated mice displays phenotypes dependent upon the infecting host [180]. This has been corroborated by more recent experiments, which demonstrate that Aβ aggregates from distinct sources have unique biophysical characteristics depending on the seeding protein [182,183,184]. While it is possible that sporadic misfolded or damaged proteins act as seeds in Alzheimer’s, this is unlikely given the steep increase in disease incidence later in life and the constant turnover of cellular proteins [185]. This steep rise in incidence mirrors the rise in incidence of CJD in individuals who have predisposing mutations [186]. It is possible that in both diseases misfolded proteins arising as a result of age‑related somatic mutation or damage to other macromolecules in single cells act as seeds for the initial protein aggregates.

### 3.3. Monogenic Disease

A list of diseases suspected to be caused by obligatory somatic mutations has been previously described [21] and subsequently updated [19,20]. We note that somatic mutation likely contributes significantly to nearly all Mendelian diseases.

We have described a series of oncogenes and tumor suppressor genes that undergo somatic mutation in cancer. These same genes can also acquire somatic mutations that result in neurocutaneous disorders or overgrowth syndromes, depending the particular cell type and developmental stage at which the mutation occurs. Mutations in *GNAQ* cause Sturge-Weber syndrome and port-wine stain birthmarks as well as uveal melanoma, as discussed above. Similarly, somatic mutations in *GNAS* can cause McCune-Albright syndrome or benign tumors such as adenomas. We next highlight several specific examples of such disorders affecting genes encoding intracellular signaling pathways (Figure 4).

Phosphatidylinositol 3-kinases (PIK3s) are lipid kinases that phosphorylate phosphatidylinositol and other phosphoinositides, catalyzing intracellular signaling pathways involving a PI3K/AKT/mTOR network (Figure 4). Somatic, mosaic, gain-of-function mutations in *PIK3CA* (OMIM *171834) are associated with several syndromes involving overgrowth of the brain or lipomatous body overgrowth [191]. These include CLOVE syndrome, megalencephaly-capillary malformation syndrome, fibroadipose hyperplasia, and hemimegalencephaly. These conditions are often characterized by early segmental overgrowth, abnormal vasculogenesis, digital anomalies, cortical brain malformations, and connective tissue dysplasia. Somatic gain-of-function mutations in *PIK3CA* are also found in a broad range of cancers (ovarian, breast, lung, stomach, colorectal, and brain). While over 100 activating mutations in *PIK3CA* are known, mutations in two domains of the protein account for 80% of cancer-associated somatic mutations, and these same sites can be mutated in overgrowth disorders [192].

Clinical presentation of Proteus syndrome (OMIM #176920) includes distorting, progressive overgrowth of various tissues including skin, skeleton, adipose, and central nervous system. In most patients it is caused by somatic mosaic mutation of *AKT1* involving c.49G > A (p.Glu17Lys) [61]. This identical mutation is associated with breast, colorectal and ovarian cancers [193]. Mutations in the homologs of *AKT1*, *AKT2* and *AKT3* are also known to cause somatic disorder. p.Glu17Lys mutations in *AKT3* cause hemimegalencephaly and other brain malformations, while the identical mutation in *AKT2* is causative for hypoglycemia [60,62,194,195].

Germline inactivating mutations in the *TSC1* gene encoding hamartin cause tuberous sclerosis 1 (OMIM #191100), while mutations in *TSC2* encoding tuberin cause tuberous sclerosis 2 (OMIM #613254). Hamartin and tuberin act as tumor suppressors by activating the GTPase function of RHEB [196]. Inactivating mutations in a single allele are sufficient to cause tuberous sclerosis. Rare somatic inactivating mutations, lack of expression of the second allele or mosaic UDP events give rise to the multiple benign tumors, tubers and macules characteristic of the disease [197,198].

Neurofibromatosis 1 (OMIM #162200) (NF1) is characterized by the occurrence of at least two (of a list of seven) features such as *café au lait* spots, cutaneous neurofibromas, Lisch nodules (hamartomas) of the iris, and inguinal freckles [199]. Clinical diagnosis requires a first-degree relative with the condition. It is inherited in an autosomal dominant manner (and is among the most common such disorders with a prevalence of 1:3000). Most cases of NF1 are caused by heterozygous loss-of-function mutations of the tumor suppressor gene encoding neurofibromin 1. Only 50% of NF1 individuals have an affected parent, with another 50% having a *de novo* mutation. Neurofibromin 1 is a negative regulator of the RAS signal transduction pathway, with loss of function mutations in neurofibromin 1 leading to RAS activation.

It is possible that mosaic variation occurring during development may result in disease across numerous tissues. One such example is somatic mutation of *IDH1* and *IDH2* that has been shown to cause Ollier disease and Maffucci syndrome. These syndromes are characterized by multiple enchondromas (benign bone tumors originating from cartilage). The causative variants for disease are typically not detectable outside of the tumors indicating that relatively few cells harbor the mutation [200].

The application of sensitive approaches for the detection of mosaicism to a smaller subset of genes based on a patient’s phenotype may increase the likelihood of finding causative variants. Jamuar* et al.* applied this approach examining two sets of previously implicated genes in 158 individuals with cerebral cortical defects. Causal mutations were found in 27 individuals, eight of who harbored the causative variant in a mosaic fashion. Notably, causal mutations were only validated at extremely high read depth (>500×) highlighting both the importance of sequence coverage for the detection of mosaic variation and the utility of targeted approaches [201].

Somatic mutations are also known to cause reversion to normal mutations in individuals with monogenic disease [22,23,25,202,203]. Revertant mosaicism occurs when cells harboring a disease-causing mutation revert* in vivo* to a wild-type allele. The disease-causing mutation could be inherited from the germline or somatic. This has been observed for heritable skin diseases such as ichthyosis with confetti (OMIM #609165) and epidermolysis bullosa (OMIM #226650) [202,204] as well as rare blood disorders such as Fanconi anemia (OMIM #227646) and severe combined immunodeficiency resulting from adenosine deaminase deficiency (OMIM #102700) [205,206]. These somatic reversions to normal events may significantly ameliorate disease symptoms if the reversion occurs early enough in development.

For many other overgrowth syndromes somatic mutations have yet to be identified. Examples include Klippel-Trenaunay-Weber syndrome (OMIM %149,000), which involves cutaneous hemangiomata and clinically resembles Sturge-Weber syndrome; and Cobb syndrome (cutaneomeningospinal angiomatosis), which involves vascular cutaneous, muscular, osseous, or other lesions of spinal segments.

### 3.4. Complex Disease

Multiple recent papers have proposed that somatic mutation may play a role in the etiology of complex disease [3,207,208]. Studies of simplex autism probands have determined that *de novo* mutations account for 2%–15% of disease incidence and that at least 30% of *de novo* mutations can be causally implicated in simplex cases [209,210,211,212]. With *de novo* mutations playing such a large role, it is likely that post-zygotic somatic variation also contributes to disease in some individuals. To date, most genetic analysis has found few genetic variants to explain complex disease incidence, suggesting the occurrence of “missing heritability” [213]. A possible model is that somatic variation occurs in conjunction with common and rare inherited variation to cause disease. While this model is not directly supported by current evidence, recent experiments indicate that it warrants investigation. One surprising result from* in situ* hybridization experiments on postmortem brain tissue is the increased presence of patches of cortical disorganization in individuals with autism relative to controls [214]. The authors note that they examined only a small subsection of the brain and therefore cortical disorganization is likely widespread in individuals with autism. Furthermore, an interesting conclusion of recent large-scale examination of exonic *de novo* mutations in simplex autism is that most *de novo* variation implicated as causal occurs opposite wild type alleles [212]. Given that large CNVs are common in neurons of the cortex [78,80], we propose a model of brain-specific somatic mutation occurring opposite inherited *de novo* or rare mutation resulting in sporadic brain-specific loss of gene function and patches of cortical disorganization.

## 4. Conclusions

While the role of somatic mosaicism in disease is currently under active investigation, it is clear that functional somatic mosaicism has a significant role in human disease. In the last decade, major advances in both cytogenetic and second-generation sequencing techniques have enabled researchers to discover causative somatic mutations for an increasing number of diseases, and driver mutations in an increasing number of cancers. Furthermore, this increased understanding of the genetic underpinnings of disease is likely to lead to improved patient outcomes in the near future.

## References

[1] Edwards J.H. (1989). Familiarity, recessivity and germline mosaicism. Ann. Hum. Genet..

[2] Hartl D.L. (1971). Recurrence risks for germinal mosaics. Am. J. Hum. Genet..

[3] Poduri A., Evrony G.D., Xuyu C., Walsh C.A. (2013). Somatic mutation, genomic variation, and neurological disease. Science.

[4] Campbell I.M., Yuan B., Robberecht C., Pfundt R., Szafranski P., McEntagart M.E., Nagamani S.C., Erez A., Bartnik M., Wisniowiecka-Kowalnik B. (2014). Parental somatic mosaicism is underrecognized and influences recurrence risk of genomic disorders. Am. J. Hum. Genet..

[5] Van der Maarel S.M., Deidda G., Lemmers R.J.L.F., van Overveld P.G.M., van der Wielen M., Hewitt J.E., Sandkuijl L., Bakker B., van Ommen G.-J.B., Padberg G.W. (2000). *De novo* facioscapulohumeral muscular dystrophy: Frequent somatic mosaicism, sex-dependent phenotype, and the role of mitotic transchromosomal repeat interaction between chromosomes 4 and 10. Am. J. Hum. Genet..

[6] Boveri T. (1929). The Origin of Malignant Tumors.

[7] Knudson A.G. (1971). Mutation and cancer: Statistical study of retinoblastoma. Proc. Natl. Acad. Sci. USA.

[8] Nowell P.C. (1976). The clonal evolution of tumor cell populations. Science.

[9] Vogelstein B., Papadopoulos N., Velculescu V.E., Zhou S., Diaz L.A., Kinzler K.W. (2013). Cancer genome landscapes. Science.

[10] McClintock B. (1951). Chromosome organization and genic expression. Cold Spring Harb. Symp. Quant. Biol..

[11] Burnet M. (1959). The Clonal Selection Theory of Acquired Immunity.

[12] Brack C., Hirama M., Lenhard-Schuller R., Tonegawa S. (1978). A complete immunoglobulin gene is created by somatic recombination. Cell.

[13] Tonegawa S. (1983). Somatic generation of antibody diversity. Nature.

[14] Krangel M.S. (2009). Mechanics of t cell receptor gene rearrangement. Curr. Opin. Immunol..

[15] Frumkin D., Wasserstrom A., Kaplan S., Feige U., Shapiro E. (2005). Genomic variability within an organism exposes its cell lineage tree. PLOS Comput. Biol..

[16] Hoeijmakers J.H.J. (2009). DNA damage, aging, and cancer. N. Engl. J. Med..

[17] Kennedy S.R., Loeb L.A., Herr A.J. (2012). Somatic mutations in aging, cancer and neurodegeneration. Mech. Ageing Dev..

[18] Jeppesen D.K., Bohr V.A., Stevnsner T. (2011). DNA repair deficiency in neurodegeneration. Prog. Neurobiol..

[19] Erickson R.P. (2010). Somatic gene mutation and human disease other than cancer: An update. Mutat. Res..

[20] Erickson R.P. (2014). Recent advances in the study of somatic mosaicism and diseases other than cancer. Curr. Opin. Genet. Dev..

[21] Erickson R.P. (2003). Somatic gene mutation and human disese other than cancer. Mutat. Res..

[22] Hirschhorn R. (2003). *In vivo* reversion to normal of inherited mutations in humans. J. Med. Genet..

[23] Jonkman M.F., Castellanos Nuijts M., van Essen A.J. (2003). Natural repair mechanisms in correcting pathogenic mutations in inherited skin disorders. Clin. Exp. Dermatol..

[24] Lai-Cheong J.E., McGrath J.A., Uitto J. (2011). Revertant mosaicism in skin: Natural gene therapy. Trends Mol. Med..

[25] Jonkman M.F. (1999). Revertant mosaicism in human genetic disorders. Am. J. Med. Genet..

[26] Happle R. (1987). Lethal genes surviving by mosaicism: A possible explanation for sporadic birth defects involving the skin. J. Am. Acad. Dermatol..

[27] Liu P., Carvalho C.M., Hastings P.J., Lupski J.R. (2012). Mechanisms for recurrent and complex human genomic rearrangements. Curr. Opin. Genet. Dev..

[28] Cimini D., Howell B., Maddox P., Khodjakov A., Degrassi F., Salmon E.D. (2001). Merotelic kinetochore orientation is a major mechanism of aneuploidy in mitotic mammalian tissue cells. J. Cell Biol..

[29] Robinson W.P. (2000). Mechanisms leading to uniparental disomy and their clinical consequences. Bioessays.

[30] Kotzot D. (2008). Complex and segmental uniparental disomy updated. J. Med. Genet..

[31] Conlin L.K., Thiel B.D., Bonnemann C.G., Medne L., Ernst L.M., Zackai E.H., Deardorff M.A., Krantz I.D., Hakonarson H., Spinner N.B. (2010). Mechanisms of mosaicism, chimerism and uniparental disomy identified by single nucleotide polymorphism array analysis. Hum. Mol. Genet..

[32] Liehr T. (2010). Cytogenetic contribution to uniparental disomy (UPD). Mol. Cytogenet..

[33] Hancks D.C., Kazazian H.H. (2012). Active human retrotransposons: Variation and disease. Curr. Opin. Genet. Dev..

[34] van den Hurk J.A., Meij I.C., Seleme M.C., Kano H., Nikopoulos K., Hoefsloot L.H., Sistermans E.A., de Wijs I.J., Mukhopadhyay A., Plomp A.S. (2007). L1 retrotransposition can occur early in human embryonic development. Hum. Mol. Genet..

[35] Lee E., Iskow R., Yang L., Gokcumen O., Haseley P., Luquette L.J., Lohr J.G., Harris C.C., Ding L., Wilson R.K. (2012). Landscape of somatic retrotransposition in human cancers. Science.

[36] Mirkin S.M. (2007). Expandable DNA repeats and human disease. Nature.

[37] Kim J.C., Mirkin S.M. (2013). The balancing act of DNA repeat expansions. Curr. Opin. Genet. Dev..

[38] McMurray C.T. (2010). Mechanisms of trinucleotide repeat instability during human development. Nat. Rev. Genet..

[39] Hellenbroich Y., Schwinger E., Zühlke C.H. (2001). Limited somatic mosaicism for friedreich’s ataxia GAA triplet repeat expansions identified by small pool PCR in blood leukocytes. Acta Neurol. Scand..

[40] Hashida H., Goto J., Suzuki T., Jeong S.-Y., Masuda N., Ooie T., Tachiiri Y., Tsuchiya H., Kanazawa I. (2001). Single cell analysis of cag repeat in brains of dentatorubral-pallidoluysian atrophy (DRPLA). J. Neurol. Sci..

[41] Kahlem P., Djian P. (2000). The expanded CAG repeat associated with juvenile Huntington disease shows a common origin of most or all neurons and glia in human cerebrum. Neurosci. Lett..

[42] Møllersen L., Rowe A.D., Larsen E., Rognes T., Klungland A. (2010). Continuous and periodic expansion of CAG repeats in Huntington’s disease R6/1 mice. PLOS Genet..

[43] Ueno S.-i., Kondoh K., Komure Y., Komure O., Kuno S., Kawai J., Hazama F., Sano A. (1995). Somatic mosaicism of CAG repeat in dentatorubral-pallidoluysian atrophy (drpla). Hum. Mol. Genet..

[44] Montermini L., Kish S.J., Jiralerspong S., Lamarche J.B., Pandolfo M. (1997). Somatic mosaicism for friedreich’s ataxia GAA triplet repeat expansions in the central nervous system. Neurology.

[45] Lindahl T., Wood R.D. (1999). Quality control by DNA repair. Science.

[46] Hoeijmakers J.H.J. (2001). Genome maintenance mechanisms for preventing cancer. Nature.

[47] Gilbert D.M. (2001). Making sense of eukaryotic DNA replication origins. Science.

[48] Gilbert D.M. (2010). Evaluating genome-scale approaches to eukaryotic DNA replication. Nat. Rev. Genet..

[49] Levy M.Z., Allsopp R.C., Futcher A.B., Greider C.W., Harley C.B. (1992). Telomere end-replication problem and cell aging. J. Mol. Biol..

[50] Van Echten-Arends J., Mastenbroek S., Sikkema-Raddatz B., Korevaar J.C., Heineman M.J., van der Veen F., Repping S. (2011). Chromosomal mosaicism in human preimplantation embryos: A systematic review. Hum. Reprod. Update.

[51] Shapiro E., Biezuner T., Linnarsson S. (2013). Single-cell sequencing-based technologies will revolutionize whole-organism science. Nat. Rev. Genet..

[52] Amat F., Lemon W., Mossing D.P., McDole K., Wan Y., Branson K., Myers E.W., Keller P.J. (2014). Fast, accurate reconstruction of cell lineages from large-scale fluorescence microscopy data. Nat. Methods.

[53] Navin N., Kendall J., Troge J., Andrews P., Rodgers L., McIndoo J., Cook K., Stepansky A., Levy D., Esposito D. (2011). Tumour evolution inferred by single-cell sequencing. Nature.

[54] Wang Y., Waters J., Leung M.L., Unruh A., Roh W., Shi X., Chen K., Scheet P., Vattathil S., Liang H. (2014). Clonal evolution in breast cancer revealed by single nucleus genome sequencing. Nature.

[55] Bolognia J.L., Orlow S.J., Glick S.A. (1994). Lines of blaschko. J. Am. Acad. Dermatol..

[56] Shirley M.D., Tang H.T., Gallione B.A., Baugher J.D., Frelin L.P., Cohen B., North P.E., Marchuk D.A., Comi A.M., Pevsner J. (2013). Sturge-weber syndrome and port-wine stains caused by somatic mutation in *GNAQ*. N. Engl. J. Med..

[57] Van Raamsdonk C.D., Griewank K.G., Crosby M.B., Garrido M.C., Vemula S., Wiesner T., Obenauf A.C., Wackernagel W., Green G., Bouvier N. (2010). Mutations in GNA11 in uveal melanoma. N. Engl. J. Med..

[58] Collins M.T., Singer F.R., Eugster E. (2012). Mccune-albright syndrome and the extraskeletal manifestations of fibrous dysplasia. Orphanet J. Rare Dis..

[59] Bastepe M., Juppner H. (2005). Gnas locus and pseudohypoparathyroidism. Horm. Res..

[60] Poduri A., Evrony G.D., Cai X., Elhosary P.C., Beroukhim R., Lehtinen M.K., Hills L.B., Heinzen E.L., Hill A., Hill R.S. (2012). Somatic activation of AKT3 causes hemispheric developmental brain malformations. Neuron.

[61] Lindhurst M.J., Sapp J.C., Teer J.K., Johnston J.J., Finn E.M., Peters K., Turner J., Cannons J.L., Bick D., Blakemore L. (2011). A mosaic activating mutation in AKT1 associated with the proteus syndrome. N. Engl. J. Med..

[62] Hussain K., Challis B., Rocha N., Payne F., Minic M., Thompson A., Daly A., Scott C., Harris J., Smillie B.J. (2011). An activating mutation of AKT2 and human hypoglycemia. Science.

[63] Jacobs K.B., Yeager M., Zhou W., Wacholder S., Wang Z., Rodriguez-Santiago B., Hutchinson A., Deng X., Liu C., Horner M.-J. (2012). Detectable clonal mosaicism and its relationship to aging and cancer. Nat. Genet..

[64] Laurie C.C., Laurie C.A., Rice K., Doheny K.F., Zelnick L.R., McHugh C.P., Ling H., Hetrick K.N., Pugh E.W., Amos C. (2012). Detectable clonal mosaicism from birth to old age and its relationship to cancer. Nat. Genet..

[65] Aghili L., Foo J., DeGregori J., De S. (2014). Patterns of somatically acquired amplifications and deletions in apparently normal tissues of ovarian cancer patients. Cell Rep..

[66] Costa T., Lambert M., Teshima I., Ray P.N., Richer C.-L., Dallaire L. (1998). Monozygotic twins with 45, X/46, XY mosaicism discordant for phenotypic sex. Am. J. Med. Genet..

[67] Fujimoto A., Boelter W.D., Sparkes R.S., Lin M.S., Battersby K. (1991). Monozygotic twins of discordant sex both with 45,X/46,X,idic(Y) mosaicism. Am. J. Med. Genet..

[68] Kaplan L., Foster R., Shen Y., Parry D.M., McMaster M.L., O’Leary M.C., Gusella J.F. (2010). Monozygotic twins discordant for neurofibromatosis 1. Am. J. Med. Genet. A.

[69] Zeng S., Patil S.R., Yankowitz J. (2003). Prenatal detection of mosaic trisomy 1q due to an unbalanced translocation in one fetus of a twin pregnancy following in vitro fertilization: A postzygotic error. Am. J. Med. Genet. A.

[70] Helderman-van den Enden A.T.J.M., Maaswinkel-Mooij P.D., Hoogendoorn E., Willemsen R., Maat-Kievit J.A., Losekoot M., Oostra B.A. (1999). Monozygotic twin brothers with the fragile X syndrome: Different CGG repeats and different mental capacities. J. Med. Genet..

[71] Piotrowski A., Bruder C.E., Andersson R., Diaz de Stahl T., Menzel U., Sandgren J., Poplawski A., von Tell D., Crasto C., Bogdan A. (2008). Somatic mosaicism for copy number variation in differentiated human tissues. Hum. Mutat..

[72] O’Huallachain M., Karczewski K.J., Weissman S.M., Urban A.E., Snyder M.P. (2012). Extensive genetic variation in somatic human tissues. Proc. Natl. Acad. Sci. USA.

[73] Abyzov A., Mariani J., Palejev D., Zhang Y., Haney M.S., Tomasini L., Ferrandino A.F., Rosenberg Belmaker L.A., Szekely A., Wilson M. (2012). Somatic copy number mosaicism in human skin revealed by induced pluripotent stem cells. Nature.

[74] Rehen S.K., McConnell M.J., Kaushal D., Kingsbury M.A., Yang A.H., Chun J. (2001). Chromosomal variation in neurons of the developing and adult mammalian nervous system. Proc. Natl. Acad. Sci. USA.

[75] Baillie J.K., Barnett M.W., Upton K.R., Gerhardt D.J., Richmond T.A., de Sapio F., Brennan P.M., Rizzu P., Smith S., Fell M. (2011). Somatic retrotransposition alters the genetic landscape of the human brain. Nature.

[76] Coufal N.G., Garcia-Perez J.L., Peng G.E., Yeo G.W., Mu Y., Lovci M.T., Morell M., O’Shea K.S., Moran J.V., Gage F.H. (2009). L1 retrotransposition in human neural progenitor cells. Nature.

[77] Muotri A.R., Chu V.T., Marchetto M.C., Deng W., Moran J.V., Gage F.H. (2005). Somatic mosaicism in neuronal precursor cells mediated by l1 retrotransposition. Nature.

[78] Cai X., Evrony G.D., Lehmann H.S., Elhosary P.C., Mehta B.K., Poduri A., Walsh C.A. (2014). Single-cell, genome-wide sequencing identifies clonal somatic copy-number variation in the human brain. Cell Rep..

[79] Evrony G.D., Cai X., Lee E., Hills L.B., Elhosary P.C., Lehmann H.S., Parker J.J., Atabay K.D., Gilmore E.C., Poduri A. (2012). Single-neuron sequencing analysis of L1 retrotransposition and somatic mutation in the human brain. Cell.

[80] McConnell M.J., Lindberg M.R., Brennand K.J., Piper J.C., Voet T., Cowing-Zitron C., Shumilina S., Lasken R.S., Vermeesch J.R., Hall I.M. (2013). Mosaic copy number variation in human neurons. Science.

[81] Kalousek D.K., Vekemans M. (1996). Confined placental mosaicism. J. Med. Genet..

[82] Kalousek D.K., Dill F.J. (1983). Chromosomal mosaicism confined to the placenta in human conceptions. Science.

[83] Taylor T.H., Gitlin S.A., Patrick J.L., Crain J.L., Wilson J.M., Griffin D.K. (2014). The origin, mechanisms, incidence and clinical consequences of chromosomal mosaicism in humans. Hum. Reprod. Update.

[84] Vanneste E., Voet T., Le Caignec C., Ampe M., Konings P., Melotte C., Debrock S., Amyere M., Vikkula M., Schuit F. (2009). Chromosome instability is common in human cleavage-stage embryos. Nat. Med..

[85] Chen E.Z., Chiu R.W.K., Sun H., Akolekar R., Chan K.C.A., Leung T.Y., Jiang P., Zheng Y.W.L., Lun F.M.F., Chan L.Y.S. (2011). Noninvasive prenatal diagnosis of fetal trisomy 18 and trisomy 13 by maternal plasma DNA sequencing. PLOS ONE.

[86] Youssoufian H., Pyeritz R.E. (2002). Mechanisms and consequences of somatic mosaicism in humans. Nat. Rev. Genet..

[87] Watson I.R., Takahashi K., Futreal P.A., Chin L. (2013). Emerging patterns of somatic mutations in cancer. Nat. Rev. Genet..

[88] Ito Y., Tanaka F., Yamamoto M., Doyu M., Nagamatsu M., Riku S., Mitsuma T., Sobue G. (1998). Somatic mosaicism of the expanded cag trinucleotide repeat in mrnas for the responsible gene of machado-joseph disease (MJD), dentatorubral-pallidoluysian atrophy (DRPLA), and spinal and bulbar muscular atrophy (SBMA). Neurochem. Res..

[89] James C.D., Carlbom E., Nordenskjold M., Collins V.P., Cavenee W.K. (1989). Mitotic recombination of chromosome 17 in astrocytomas. Proc. Natl. Acad. Sci. USA.

[90] Kleczkowska A., Fryns J.P., Van den Berghe H. (1990). On the variable effect of mosaic normal/balanced chromosomal rearrangements in man. J. Med. Genet..

[91] Kotzot D., Schmitt S., Bernasconi F., Robinson W.P., Lurie I.W., Ilyina H., Méhes K., Hamel B.C.J., Otten B.J., Hergersberg M. (1995). Uniparental disomy 7 in silver-russell syndrome and primordial growth retardation. Hum. Mol. Genet..

[92] Rodríguez-Santiago B., Malats N., Rothman N., Armengol L., Garcia-Closas M., Kogevinas M., Villa O., Hutchinson A., Earl J., Marenne G. (2010). Mosaic uniparental disomies and aneuploidies as large structural variants of the human genome. Am. J. Hum. Genet..

[93] Slatter R.E., Elliott M., Welham K., Carrera M., Schofield P.N., Barton D.E., Maher E.R. (1994). Mosaic uniparental disomy in beckwith-wiedemann syndrome. J. Med. Genet..

[94] Zori R.T., Gray B.A., Bent-Williams A., Driscoll D.J., Williams C.A., Zackowski J.L. (1993). Preaxial acrofacial dysostosis (nager syndrome) associated with an inherited and apparently balanced X;9 translocation: Prenatal and postnatal late replication studies. Am. J. Med. Genet..

[95] Walsh C., Cepko C.L. (1992). Widespread dispersion of neuronal clones across functional regions of the cerebral cortex. Science.

[96] Pleasure S.J., Anderson S., Hevner R., Bagri A., Marin O., Lowenstein D.H., Rubenstein J.L. (2000). Cell migration from the ganglionic eminences is required for the development of hippocampal gabaergic interneurons. Neuron.

[97] Hohn A., Leibrock J., Bailey K., Barde Y.-A. (1990). Identification and characterization of a novel member of the nerve growth factor/brain-derived neurotrophic factor family. Nature.

[98] Leibrock J., Lottspeich F., Hohn A., Hofer M., Hengerer B., Masiakowski P., Thoenen H., Barde Y.-A. (1989). Molecular cloning and expression of brain-derived neurotrophic factor. Nature.

[99] Levi-Montalcini R. (1964). Growth control of nerve cells by a protein factor and its antiserum: Discovery of this factor may provide new leads to understanding of some neurogenetic processes. Science.

[100] Kurek K.C., Luks V.L., Ayturk U.M., Alomari A.I., Fishman S.J., Spencer S.A., Mulliken J.B., Bowen M.E., Yamamoto G.L., Kozakewich H.P. (2012). Somatic mosaic activating mutations in PIK3CA cause cloves syndrome. Am. J. Hum. Genet..

[101] Li H. (2014). Toward better understanding of artifacts in variant calling from high-coverage samples. Bioinformatics.

[102] Gerstung M., Papaemmanuil E., Campbell P.J. (2014). Subclonal variant calling with multiple samples and prior knowledge. Bioinformatics.

[103] Crotwell P.L., Hoyme H.E. (2012). Advances in whole-genome genetic testing: From chromosomes to microarrays. Curr. Probl. Pediatr. Adolesc. Health Care.

[104] Bushman D.M., Chun J. (2013). The genomically mosaic brain: Aneuploidy and more in neural diversity and disease. Semin. Cell Dev. Biol..

[105] Notini A.J., Craig J.M., White S.J. (2008). Copy number variation and mosaicism. Cytogenet. Genome Res..

[106] Vorsanova S.G., Yurov Y.B., Iourov I.Y. (2010). Human interphase chromosomes: A review of available molecular cytogenetic technologies. Mol. Cytogenet..

[107] Imataka G., Arisaka O. (2012). Chromosome analysis using spectral karyotyping (sky). Cell Biochem. Biophys..

[108] Kallioniemi A., Kallioniemi O.P., Sudar D., Rutovitz D., Gray J.W., Waldman F., Pinkel D. (1992). Comparative genomic hybridization for molecular cytogenetic analysis of solid tumors. Science.

[109] Pinkel D., Albertson D.G. (2005). Array comparative genomic hybridization and its applications in cancer. Nat. Genet..

[110] Alkan C., Coe B.P., Eichler E.E. (2011). Genome structural variation discovery and genotyping. Nat. Rev. Genet..

[111] Bignell G.R., Huang J., Greshock J., Watt S., Butler A., West S., Grigorova M., Jones K.W., Wei W., Stratton M.R. (2004). High-resolution analysis of DNA copy number using oligonucleotide microarrays. Genome Res..

[112] Mohr S., Leikauf G.D., Keith G., Rihn B.H. (2002). Microarrays as cancer keys: An array of possibilities. J. Clin. Oncol..

[113] Baugher J.D., Baugher B.D., Shirley M.D., Pevsner J. (2013). Sensitive and specific detection of mosaic chromosomal abnormalities using the parent-of-origin-based detection (POD) method. BMC Genom..

[114] Leek J.T., Scharpf R.B., Bravo H.C., Simcha D., Langmead B., Johnson W.E., Geman D., Baggerly K., Irizarry R.A. (2010). Tackling the widespread and critical impact of batch effects in high-throughput data. Nat. Rev. Genet..

[115] Chen G.K., Chang X., Curtis C., Wang K. (2013). Precise inference of copy number alterations in tumor samples from SNP arrays. Bioinformatics.

[116] Li A., Liu Z., Lezon-Geyda K., Sarkar S., Lannin D., Schulz V., Krop I., Winer E., Harris L., Tuck D. (2011). GPHMM: An integrated hidden Markov model for identification of copy number alteration and loss of heterozygosity in complex tumor samples using whole genome SNP arrays. Nucleic Acids Res..

[117] Liu Z., Li A., Schulz V., Chen M., Tuck D. (2010). Mixhmm: Inferring copy number variation and allelic imbalance using SNP arrays and tumor samples mixed with stromal cells. PLOS ONE.

[118] Rancoita P., Hutter M., Bertoni F., Kwee I. (2010). An integrated bayesian analysis of LOH and copy number data. BMC Bioinform..

[119] Cock P.J., Fields C.J., Goto N., Heuer M.L., Rice P.M. (2010). The sanger FASTQ file format for sequences with quality scores, and the solexa/illumina FASTQ variants. Nucleic Acids Res..

[120] Yoon S., Xuan Z., Makarov V., Ye K., Sebat J. (2009). Sensitive and accurate detection of copy number variants using read depth of coverage. Genome Res..

[121] Koboldt D.C., Zhang Q., Larson D.E., Shen D., McLellan M.D., Lin L., Miller C.A., Mardis E.R., Ding L., Wilson R.K. (2012). Varscan 2: Somatic mutation and copy number alteration discovery in cancer by exome sequencing. Genome Res..

[122] Ng S.B., Turner E.H., Robertson P.D., Flygare S.D., Bigham A.W., Lee C., Shaffer T., Wong M., Bhattacharjee A., Eichler E.E. (2009). Targeted capture and massively parallel sequencing of 12 human exomes. Nature.

[123] Choi M., Scholl U.I., Ji W., Liu T., Tikhonova I.R., Zumbo P., Nayir A., Bakkaloğlu A., Özen S., Sanjad S. (2009). Genetic diagnosis by whole exome capture and massively parallel DNA sequencing. Proc. Natl. Acad. Sci..

[124] Hodges E., Xuan Z., Balija V., Kramer M., Molla M.N., Smith S.W., Middle C.M., Rodesch M.J., Albert T.J., Hannon G.J. (2007). Genome-wide in situ exon capture for selective resequencing. Nat. Genet..

[125] Larson D.E., Harris C.C., Chen K., Koboldt D.C., Abbott T.E., Dooling D.J., Ley T.J., Mardis E.R., Wilson R.K., Ding L. (2012). Somaticsniper: Identification of somatic point mutations in whole genome sequencing data. Bioinformatics.

[126] Roth A., Ding J., Morin R., Crisan A., Ha G., Giuliany R., Bashashati A., Hirst M., Turashvili G., Oloumi A. (2012). Jointsnvmix: A probabilistic model for accurate detection of somatic mutations in normal/tumour paired next-generation sequencing data. Bioinformatics.

[127] Saunders C.T., Wong W.S., Swamy S., Becq J., Murray L.J., Cheetham R.K. (2012). Strelka: Accurate somatic small-variant calling from sequenced tumor-normal sample pairs. Bioinformatics.

[128] Cibulskis K., Lawrence M.S., Carter S.L., Sivachenko A., Jaffe D., Sougnez C., Gabriel S., Meyerson M., Lander E.S., Getz G. (2013). Sensitive detection of somatic point mutations in impure and heterogeneous cancer samples. Nature Biotechnol..

[129] Li H., Handsaker B., Wysoker A., Fennell T., Ruan J., Homer N., Marth G., Abecasis G., Durbin R. (2009). The sequence alignment/map format and samtools. Bioinformatics.

[130] Amarasinghe K.C., Li J., Halgamuge S.K. (2013). Convex: Copy number variation estimation in exome sequencing data using HMM. BMC Bioinform..

[131] Boeva V., Popova T., Bleakley K., Chiche P., Cappo J., Schleiermacher G., Janoueix-Lerosey I., Delattre O., Barillot E. (2012). Control-freec: A tool for assessing copy number and allelic content using next-generation sequencing data. Bioinformatics.

[132] Layer R.M., Chiang C., Quinlan A.R., Hall I.M. (2014). Lumpy: A probabilistic framework for structural variant discovery. Genome Biol..

[133] Chen M., Gunel M., Zhao H. (2013). Somatica: Identifying, characterizing and quantifying somatic copy number aberrations from cancer genome sequencing data. PLOS ONE.

[134] Ding L., Wendl M.C., McMichael J.F., Raphael B.J. (2014). Expanding the computational toolbox for mining cancer genomes. Nat. Rev. Genet..

[135] Yadav V.K., De S. (2014). An assessment of computational methods for estimating purity and clonality using genomic data derived from heterogeneous tumor tissue samples. Brief. Bioinform..

[136] Dean F.B., Hosono S., Fang L., Wu X., Faruqi A.F., Bray-Ward P., Sun Z., Zong Q., Du Y., Du J. (2002). Comprehensive human genome amplification using multiple displacement amplification. Proc. Natl. Acad. Sci. USA.

[137] Hosono S., Faruqi A.F., Dean F.B., Du Y., Sun Z., Wu X., Du J., Kingsmore S.F., Egholm M., Lasken R.S. (2003). Unbiased whole-genome amplification directly from clinical samples. Genome Res..

[138] Pugh T.J., Delaney A.D., Farnoud N., Flibotte S., Griffith M., Li H.I., Qian H., Farinha P., Gascoyne R.D., Marra M.A. (2008). Impact of whole genome amplification on analysis of copy number variants. Nucleic Acids Res..

[139] Baslan T., Kendall J., Rodgers L., Cox H., Riggs M., Stepansky A., Troge J., Ravi K., Esposito D., Lakshmi B. (2012). Genome-wide copy number analysis of single cells. Nat. Protoc..

[140] Gundry M., Li W., Maqbool S.B., Vijg J. (2012). Direct, genome-wide assessment of DNA mutations in single cells. Nucleic Acids Res..

[141] Hou Y., Song L., Zhu P., Zhang B., Tao Y., Xu X., Li F., Wu K., Liang J., Shao D. (2012). Single-cell exome sequencing and monoclonal evolution of a JAK2-negative myeloproliferative neoplasm. Cell.

[142] Xu X., Hou Y., Yin X., Bao L., Tang A., Song L., Li F., Tsang S., Wu K., Wu H. (2012). Single-cell exome sequencing reveals single-nucleotide mutation characteristics of a kidney tumor. Cell.

[143] Sjoblom T., Jones S., Wood L.D., Parsons D.W., Lin J., Barber T.D., Mandelker D., Leary R.J., Ptak J., Silliman N. (2006). The consensus coding sequences of human breast and colorectal cancers. Science.

[144] Stephens P.J., Tarpey P.S., Davies H., van Loo P., Greenman C., Wedge D.C., Nik-Zainal S., Martin S., Varela I., Bignell G.R. (2012). The landscape of cancer genes and mutational processes in breast cancer. Nature.

[145] Stratton M.R., Campbell P.J., Futreal P.A. (2009). The cancer genome. Nature.

[146] Hanahan D., Weinberg R.A. (2011). Hallmarks of cancer: The next generation. Cell.

[147] McCubrey J.A., Steelman L.S., Chappell W.H., Abrams S.L., Montalto G., Cervello M., Nicoletti F., Fagone P., Malaponte G., Mazzarino M.C. (2012). Mutations and deregulation of Ras/Raf/MEK/ERK and PI3K/PTEN/Akt/mTOR cascades which alter therapy response. Oncotarget.

[148] Downward J. (2003). Targeting RAS signalling pathways in cancer therapy. Nat. Rev. Cancer.

[149] Liaw D., Marsh D.J., Li J., Dahia P.L.M., Wang S.I., Zheng Z., Bose S., Call K.M., Tsou H.C., Peacoke M. (1997). Germline mutations of the pten gene in cowden disease, an inherited breast and thyroid cancer syndrome. Nat. Genet..

[150] Malkin D., Li F., Strong L., Fraumeni J., Nelson C., Kim D., Kassel J., Gryka M., Bischoff F., Tainsky M. (1990). Germ line p53 mutations in a familial syndrome of breast cancer, sarcomas, and other neoplasms. Science.

[151] Morin P.J., Sparks A.B., Korinek V., Barker N., Clevers H., Vogelstein B., Kinzler K.W. (1997). Activation of β-catenin-TCF signaling in colon cancer by mutations in β-catenin or APC. Science.

[152] Wooster R., Bignell G., Lancaster J., Swift S., Seal S., Mangion J., Collins N., Gregory S., Gumbs C., Micklem G. (1995). Identification of the breast cancer susceptibility gene BRCA2. Nature.

[153] Moynahan M.E., Chiu J.W., Koller B.H., Jasin M. (1999). Brca1 controls homology-directed DNA repair. Mol. Cell.

[154] Miyaki M., Konishi M., Tanaka K., Kikuchi-Yanoshita R., Muraoka M., Yasuno M., Igari T., Koike M., Chiba M., Mori T. (1997). Germline mutation of MSH6 as the cause of hereditary nonpolyposis colorectal cancer. Nat. Genet..

[155] Cleaver J.E. (1968). Defective repair replication of DNA in xeroderma pigmentosum. Nature.

[156] Govindan R., Ding L., Griffith M., Subramanian J., Dees N.D., Kanchi K.L., Maher C.A., Fulton R., Fulton L., Wallis J. (2012). Genomic landscape of non-small cell lung cancer in smokers and never-smokers. Cell.

[157] Forbes S.A., Bindal N., Bamford S., Cole C., Kok C.Y., Beare D., Jia M., Shepherd R., Leung K., Menzies A. (2011). Cosmic: Mining complete cancer genomes in the catalogue of somatic mutations in cancer. Nucleic Acids Res..

[158] Collins F.S., Barker A.D. (2007). Mapping the cancer genome. Pinpointing the genes involved in cancer will help chart a new course across the complex landscape of human malignancies. Sci. Am..

[159] Debeljak M., Freed D.N., Welch J.A., Haley L., Beierl K., Iglehart B.S., Pallavajjala A., Gocke C.D., Leffell M.S., Lin M.-T. (2014). Haplotype counting by next-generation sequencing for ultrasensitive human DNA detection. J. Mol. Diagn..

[160] Armitage P., Doll R. (1954). The age distribution of cancer and a multi-stage theory of carcinogenesis. Br. J. Cancer.

[161] Szilard L. (1959). On the nature of the aging process. Proc. Natl. Acad. Sci. USA.

[162] Curtis H.J. (1963). Biological mechanisms underlying the aging process. Science.

[163] Goldsby R.E., Hays L.E., Chen X., Olmsted E.A., Slayton W.B., Spangrude G.J., Preston B.D. (2002). High incidence of epithelial cancers in mice deficient for DNA polymerase δ proofreading. Proc. Natl. Acad. Sci. USA.

[164] Goldsby R.E., Lawrence N.A., Hays L.E., Olmsted E.A., Chen X., Singh M., Preston B.D. (2001). Defective DNA polymerase-[delta] proofreading causes cancer susceptibility in mice. Nat. Med..

[165] Albertson T.M., Ogawa M., Bugni J.M., Hays L.E., Chen Y., Wang Y., Treuting P.M., Heddle J.A., Goldsby R.E., Preston B.D. (2009). DNA polymerase ε and δ proofreading suppress discrete mutator and cancer phenotypes in mice. Proc. Natl. Acad. Sci. USA.

[166] Vermulst M., Bielas J.H., Kujoth G.C., Ladiges W.C., Rabinovitch P.S., Prolla T.A., Loeb L.A. (2007). Mitochondrial point mutations do not limit the natural lifespan of mice. Nat. Genet..

[167] Trifunovic A., Wredenberg A., Falkenberg M., Spelbrink J.N., Rovio A.T., Bruder C.E., Bohlooly-Y M., Gidlof S., Oldfors A., Wibom R. (2004). Premature ageing in mice expressing defective mitochondrial DNA polymerase. Nature.

[168] Marteijn J.A., Lans H., Vermeulen W., Hoeijmakers J.H.J. (2014). Understanding nucleotide excision repair and its roles in cancer and ageing. Nat. Rev. Mol. Cell. Biol..

[169] Mohaghegh P., Hickson I.D. (2001). DNA helicase deficiencies associated with cancer predisposition and premature ageing disorders. Hum. Mol. Genet..

[170] Hanks S., Coleman K., Reid S., Plaja A., Firth H., Fitzpatrick D., Kidd A., Mehes K., Nash R., Robin N. (2004). Constitutional aneuploidy and cancer predisposition caused by biallelic mutations in bub1b. Nat. Genet..

[171] Date H., Onodera O., Tanaka H., Iwabuchi K., Uekawa K., Igarashi S., Koike R., Hiroi T., Yuasa T., Awaya Y. (2001). Early-onset ataxia with ocular motor apraxia and hypoalbuminemia is caused by mutations in a new HIT superfamily gene. Nat. Genet..

[172] Moreira M.-C., Barbot C., Tachi N., Kozuka N., Uchida E., Gibson T., Mendonca P., Costa M., Barros J., Yanagisawa T. (2001). The gene mutated in ataxia-ocular apraxia 1 encodes the new HIT/Zn-finger protein aprataxin. Nat. Genet..

[173] Niedernhofer L.J. (2008). Tissue-specific accelerated aging in nucleotide excision repair deficiency. Mech. Ageing Dev..

[174] Monnat R.J. (2010). Human RECQ helicases: Roles in DNA metabolism, mutagenesis and cancer biology. Semin. Cancer Biol..

[175] Burdick D., Soreghan B., Kwon M., Kosmoski J., Knauer M., Henschen A., Yates J., Cotman C., Glabe C. (1992). Assembly and aggregation properties of synthetic Alzheimer’s A4/beta amyloid peptide analogs. J. Biol. Chem..

[176] Goldfarb L.G., Brown P., McCombie W.R., Goldgaber D., Swergold G.D., Wills P.R., Cervenakova L., Baron H., Gibbs C.J., Gajdusek D.C. (1991). Transmissible familial Creutzfeldt-Jakob disease associated with five, seven, and eight extra octapeptide coding repeats in the PRNP gene. Proc. Natl. Acad. Sci. USA.

[177] Beck J.A., Poulter M., Campbell T.A., Uphill J.B., Adamson G., Geddes J.F., Revesz T., Davis M.B., Wood N.W., Collinge J. (2004). Somatic and germline mosaicism in sporadic early-onset alzheimer’s disease. Hum. Mol. Genet..

[178] Alzualde A., Moreno F., Martínez-Lage P., Ferrer I., Gorostidi A., Otaegui D., Blázquez L., Atares B., Cardoso S., Martínez de Pancorbo M. (2010). Somatic mosaicism in a case of apparently sporadic Creutzfeldt-Jakob disease carrying a *de novo* D178n mutation in the PRNP gene. Am. J. Med. Genet. B Neuropsychiatr. Genet..

[179] Eikelenboom P., Bate C., Van Gool W.A., Hoozemans J.J.M., Rozemuller J.M., Veerhuis R., Williams A. (2002). Neuroinflammation in alzheimer’s disease and prion disease. Glia.

[180] Meyer-Luehmann M., Coomaraswamy J., Bolmont T., Kaeser S., Schaefer C., Kilger E., Neuenschwander A., Abramowski D., Frey P., Jaton A.L. (2006). Exogenous induction of cerebral β-amyloidogenesis is governed by agent and host. Science.

[181] Kane M.D., Lipinski W.J., Callahan M.J., Bian F., Durham R.A., Schwarz R.D., Roher A.E., Walker L.C. (2000). Evidence for seeding of beta-amyloid by intracerebral infusion of alzheimer brain extracts in beta-amyloid precursor protein-transgenic mice. J. Neurosci..

[182] Lu J.-X., Qiang W., Yau W.-M., Schwieters C.D., Meredith S.C., Tycko R. (2013). Molecular structure of β-amyloid fibrils in alzheimer’s disease brain tissue. Cell.

[183] Stöhr J., Condello C., Watts J.C., Bloch L., Oehler A., Nick M., DeArmond S.J., Giles K., DeGrado W.F., Prusiner S.B. (2014). Distinct synthetic aβ prion strains producing different amyloid deposits in bigenic mice. Proc. Natl. Acad. Sci..

[184] Watts J.C., Condello C., Stöhr J., Oehler A., Lee J., DeArmond S.J., Lannfelt L., Ingelsson M., Giles K., Prusiner S.B. (2014). Serial propagation of distinct strains of aβ prions from alzheimer’s disease patients. Proc. Natl. Acad. Sci. USA.

[185] Ott A., Breteler M.M.B., van Harskamp F., Claus J.J., van der Cammen T.J.M., Grobbee D.E., Hofman A. (1995). Prevalence of alzheimer’s disease and vascular dementia: Association with education. The Rotterdam study. BMJ.

[186] Chapman J., Ben-Israel J., Goldhammer Y., Korczyn A.D. (1994). The risk of developing creutzfeldt-jakob disease in subjects with the PRNP gene codon 200 point mutation. Neurology.

[187] Fruman D.A., Rommel C. (2014). PI3K and cancer: Lessons, challenges and opportunities. Nat. Rev. Drug Discov..

[188] Hay N. (2005). The AKT-mtor tango and its relevance to cancer. Cancer Cell.

[189] Rozengurt E. (2007). Mitogenic signaling pathways induced by G protein-coupled receptors. J. Cell. Physiol..

[190] Dorsam R.T., Gutkind J.S. (2007). G-protein-coupled receptors and cancer. Nat. Rev. Cancer.

[191] Mirzaa G., Conaway R., Graham J.M., Dobyns W.B., Pagon R.A., Adam M.P., Ardinger H.H. (2013). PIK3CA-related segmental overgrowth. GeneReviews.

[192] Samuels Y., Wang Z., Bardelli A., Silliman N., Ptak J., Szabo S., Yan H., Gazdar A., Powell S.M., Riggins G.J. (2004). High frequency of mutations of the PIK3CA gene in human cancers. Science.

[193] Carpten J.D., Faber A.L., Horn C., Donoho G.P., Briggs S.L., Robbins C.M., Hostetter G., Boguslawski S., Moses T.Y., Savage S. (2007). A transforming mutation in the pleckstrin homology domain of AKT1 in cancer. Nature.

[194] Riviere J.B., Mirzaa G.M., O'Roak B.J., Beddaoui M., Alcantara D., Conway R.L., St-Onge J., Schwartzentruber J.A., Gripp K.W., Nikkel S.M. (2012). *De novo* germline and postzygotic mutations in AKT3, PIK3R2 and PIK3CA cause a spectrum of related megalencephaly syndromes. Nat. Genet..

[195] Lee J.H., Huynh M., Silhavy J.L., Kim S., Dixon-Salazar T., Heiberg A., Scott E., Bafna V., Hill K.J., Collazo A. (2012). *De novo* somatic mutations in components of the PI3K-AKT3-mtor pathway cause hemimegalencephaly. Nat. Genet..

[196] Zhang Y., Gao X., Saucedo L.J., Ru B., Edgar B.A., Pan D. (2003). RHEB is a direct target of the tuberous sclerosis tumour suppressor proteins. Nat. Cell. Biol..

[197] Curatolo P., Bombardieri R., Jozwiak S. (2008). Tuberous sclerosis. Lancet.

[198] Henske E.P., Wessner L.L., Golden J., Scheithauer B.W., Vortmeyer A.O., Zhuang Z., Klein-Szanto A.J., Kwiatkowski D.J., Yeung R.S. (1997). Loss of tuberin in both subependymal giant cell astrocytomas and angiomyolipomas supports a two-hit model for the pathogenesis of tuberous sclerosis tumors. Am. J. Pathol..

[199] Tsang E., Birch P., Friedman J.M. (2012). Valuing gene testing in children with possible neurofibromatosis 1. Clin. Genet..

[200] Pansuriya T.C., van Eijk R., d’Adamo P., van Ruler M.A., Kuijjer M.L., Oosting J., Cleton-Jansen A.M., van Oosterwijk J.G., Verbeke S.L., Meijer D. (2011). Somatic mosaic IDH1 and IDH2 mutations are associated with enchondroma and spindle cell hemangioma in ollier disease and maffucci syndrome. Nat. Genet..

[201] Jamuar S.S., Lam A.T., Kircher M., D’Gama A.M., Wang J., Barry B.J., Zhang X., Hill R.S., Partlow J.N., Rozzo A. (2014). Somatic mutations in cerebral cortical malformations. N. Engl. J. Med..

[202] Choate K.A., Lu Y., Zhou J., Choi M., Elias P.M., Farhi A., Nelson-Williams C., Crumrine D., Williams M.L., Nopper A.J. (2010). Mitotic recombination in patients with ichthyosis causes reversion of dominant mutations in KRT10. Science.

[203] Pasmooij A.M., Jonkman M.F., Uitto J. (2012). Revertant mosaicism in heritable skin diseases: Mechanisms of natural gene therapy. Discov. Med..

[204] Pasmooij A.M., Pas H.H., Bolling M.C., Jonkman M.F. (2007). Revertant mosaicism in junctional epidermolysis bullosa due to multiple correcting second-site mutations in LAMB3. J. Clin. Invest..

[205] Hirschhorn R., Yang D.R., Puck J.M., Huie M.L., Jiang C.K., Kurlandsky L.E. (1996). Spontaneous *in vivo* reversion to normal of an inherited mutation in a patient with adenosine deaminase deficiency. Nat. Genet..

[206] Soulier J., Leblanc T., Larghero J., Dastot H., Shimamura A., Guardiola P., Esperou H., Ferry C., Jubert C., Feugeas J.-P. (2005). Detection of somatic mosaicism and classification of fanconi anemia patients by analysis of the FA/BRCA pathway. Blood.

[207] De S. (2011). Somatic mosaicism in healthy human tissues. Trends Genet..

[208] Insel T.R. (2014). Brain somatic mutations: The dark matter of psychiatric genetics [quest]. Mol. Psychiatry.

[209] Krumm N., O’Roak B.J., Shendure J., Eichler E.E. (2014). A *de novo* convergence of autism genetics and molecular neuroscience. Trends Neurosci..

[210] Gaugler T., Klei L., Sanders S.J., Bodea C.A., Goldberg A.P., Lee A.B., Mahajan M., Manaa D., Pawitan Y., Reichert J. (2014). Most genetic risk for autism resides with common variation. Nat. Genet..

[211] De Rubeis S., He X., Goldberg A.P., Poultney C.S., Samocha K., Ercument Cicek A., Kou Y., Liu L., Fromer M., Walker S. (2014). Synaptic, transcriptional and chromatin genes disrupted in autism. Nature.

[212] Iossifov I., O’Roak B.J., Sanders S.J., Ronemus M., Krumm N., Levy D., Stessman H.A., Witherspoon K.T., Vives L., Patterson K.E. (2014). The contribution of de novo coding mutations to autism spectrum disorder. Nature.

[213] Manolio T.A., Collins F.S., Cox N.J., Goldstein D.B., Hindorff L.A., Hunter D.J., McCarthy M.I., Ramos E.M., Cardon L.R., Chakravarti A. (2009). Finding the missing heritability of complex diseases. Nature.

[214] Stoner R., Chow M.L., Boyle M.P., Sunkin S.M., Mouton P.R., Roy S., Wynshaw-Boris A., Colamarino S.A., Lein E.S., Courchesne E. (2014). Patches of disorganization in the neocortex of children with autism. N. Engl. J. Med..

